# Survival of the Sawfly *Athalia rosae* Upon Infection by an Entomopathogenic Fungus and in Relation to Clerodanoid Uptake

**DOI:** 10.3389/fphys.2021.637617

**Published:** 2021-03-24

**Authors:** Caroline Zanchi, Lai Ka Lo, Reshma R, Isabel Moritz, Joachim Kurtz, Caroline Müller

**Affiliations:** ^1^Animal Evolutionary Ecology Group, Institute for Evolution and Biodiversity, University of Münster, Münster, Germany; ^2^Department of Chemical Ecology, Faculty of Biology, Bielefeld University, Bielefeld, Germany

**Keywords:** *Athalia rosae*, *Beauveria bassiana*, *Ajuga reptans*, clerodanoids, pharmacophagy, phytochemicals, chemical ecology

## Abstract

Larvae of the turnip sawfly *Athalia rosae* are a pest of Brassicacae plants, as their feeding can cause defoliation of various crops of economic importance. The larvae and the adults of this sawfly species are known to take up different classes of chemical compounds from their respective host plants, with potentially deterrent functions against predators. In addition, compounds taken up by the adults, the clerodanoids, are known for their antimicrobial activity. These features could be a challenge to biocontrol strategies. Several natural enemies of *A. rosae* have been identified, targeting larval and pupal stages of *A. rosae*, which could potentially be used as biocontrol agents. However, targeting the adult stage of a larval pest in addition to targeting the juvenile stages may improve population control. In this study, we ask whether a strain of the entomopathogenic fungus *Beauveria bassiana* shows biological activity against *A. rosae* adults. We also investigate whether the behavior of clerodanoid uptake by the adults, which is commonly found, affects their survival in response to a *B. bassiana* exposure. We found a clear dose-response relationship, i.e., with increasing fungal conidia concentrations survival of *A. rosae* decreased. However, there was only a low incidence of mycelial growth and sporulation from *A. rosae* cadavers, indicating that either the fungus is not successfully developing inside this host, or it is not able to re-emerge from it. Clerodanoid uptake decreased the survival of healthy adults; however, it did not increase their survival to *B. bassiana*. Our results revealed that this strain of *B. bassiana* if applied alone is probably not suitable for biocontrol of this sawfly species, because *A. rosae* showed a high baseline resistance against this fungus. The behavior of clerodanoid uptake is unlikely to have evolved as a defense against this entomopathogenic fungus.

## Introduction

Insect herbivores can cause tremendous losses in agriculture, when feeding as larvae or adults on diverse plant parts of crops (Zalucki et al., 2012; Sharma et al., 2017). Natural enemies of insect pests are used as effective control agents against adult as well as juvenile stages of their herbivorous hosts (Kuhar et al., 2000; Laznik et al., 2010; Boivin et al., 2012). However, most examples of biological pest control involve targeting juvenile stages of insect pests. For example, the effectiveness of the most widely used biopesticide to date, *Bacillus thuringiensis*, decreases with increasing developmental stage of the insect host (Glare and O’Callaghan, 2000). Likewise, baculoviruses, applied mainly against lepidopteran pest species, target larval hosts (Inceoglu et al., 2001). In many cases, juvenile stages might be more accessible to a biocontrol agent, since they are the stages which aggregate in high densities in crop fields, and have a high feeding rate (Ohnesorge, 1979; Ferro et al., 1983). Manipulating the adult population has been shown to be an effective method of reducing the damage to crops (Hunt and Vernon, 2001; Cotes et al., 2018). However, adults are often the dispersing stage and might be harder to target (Vallat and Dorn, 2005; Lombaert et al., 2006). Another obstacle to the use of parasites and pathogens against adult insect pests is that several microbial control agents, such as *B. thuringiensis* and baculoviruses, need to be taken up orally by species which might not feed in the adult stage or feed on a food source which might not be a suitable vector for a biocontrol agent (Glare and O’Callaghan, 2000), preventing the uptake of oral pathogens.

This setback is circumvented by entomopathogenic fungi, which infect their host by penetrating through its cuticle. In the case of *Beauveria bassiana* (Ascomycota: Hypocreales), the conidia attach, germinate and penetrate the cuticle of a broad range of insect hosts (Ortiz-Urquiza and Keyhani, 2013). When the hemolymph of the host is reached, the fungus differentiates into yeast-like cells called blastospores, which proliferate in the insect’s body cavity and use its nutrients. After host death, hyphae germinate, colonize host tissues, catabolize their remaining nutrients and breach the cuticle from the inside. After colonizing the insect surface and if the environmental conditions allow it, conidia will sporulate and complete the life-cycle of the fungus (Ortiz-Urquiza and Keyhani, 2013; Pedrini et al., 2013). The ease of production of *B. bassiana* on an industrial scale (Santa et al., 2005) as well as the fact that this is a necrotrophic parasite which needs to kill its host to complete its life-cycle made it a microorganism of interest in the context of biological control and integrated pest management (Lacey et al., 2015). However, its application in the field faces several challenges: Its speed of killing is sometimes considered as too slow to prevent significant damage to the crops by feeding insects (Inglis et al., 2001), and its lethality might also decrease as a result of environmental conditions (Rangel et al., 2008) and host defenses (Maistrou et al., 2018). Insect behavior can be an important line of defense against entomopathogenic fungi (Bonadies et al., 2019) including *B. bassiana* (Meyling and Pell, 2006), which might further jeopardize the success of biocontrol strategies.

The turnip sawfly, *Athalia rosae* (Hymenoptera: Tenthredinidae), is a feeding specialist on plants of Brassicaceae. The larvae feed on leaves and flowers (Bandeili and Müller, 2010) and can cause serious losses on crops of economic importance (Sawa et al., 1989), as they often occur in high densities, leading to complete defoliation (Riggert, 1939). The control of this pest includes the use of insecticides for coating seeds of Brassicacae, as well as for later pulverization in the field (Daniel and Ion, 2019). As an alternative or complement to chemical pesticides, several natural enemies of *A. rosae* have been described, including nematodes and insect parasitoids (ichneumonids, tachinids, and chalcidids), which exclusively infest the larval stages (Riggert, 1939). The adults of *A. rosae* feed on nectar of various plants, mostly belonging to the Apiaceae. In addition, adult males and females visit different plants of the Lamiales, on which they do not cause any visible damage but take up *neo*-cleradane diterpenoids (in the following called clerodanoids; Nishida et al., 2004). These compounds act as feeding stimulants (Opitz et al., 2012), but also affect the mating behavior, making particularly females more successful once they have had access to clerodanoids (Amano et al., 1999). Moreover, adults that have incorporated these compounds are better protected against diverse predators and seem to be bitter tasting (Nishida and Fukami, 1990). Thus, adults are pharmacophagous, as they take up these compounds for purposes other than nutrition, a behavior also called self-medication (Abbott, 2014). Clerodanoids isolated from different Lamiaceae are known to have antimicrobial activity *in vitro* (Bozov et al., 2015). However, it is to our knowledge unknown yet whether exposure to clerodanoids *via* uptake from leaves can also act antimicrobial *in vivo*.

Efforts to control the populations of *A. rosae* focus on egg and larval stages, probably because of their ease of collection in the field (Sáringer et al., 1996; Kamangar et al., 2010). However, controlling adults while they aggregate on plants for clerodanoid uptake and reproduction may reduce the number of ovipositing adult females on crop fields. Moreover, it is yet unknown whether entomopathogenic fungi could be an option for the control of *A. rosae* populations. In this study, we investigated whether *B. bassiana* could be a prospective biocontrol agent against adult *A. rosae*. Additionally, we investigated whether the lethality of the fungus is influenced by the behavior of clerodanoid uptake by the sawflies.

## Materials and Methods

### Insect Maintenance

Adults of the sawfly *A. rosae* were collected from Apiaceae in the surroundings of Bielefeld, Germany, and reared in the laboratory for several generations. Adults were provided with potted host plants of *Sinapis alba* for oviposition. Larvae were offered *S. alba* and *Brassica rapa* var. *pekinensis* for feeding. The host plants were grown in the greenhouse (about 20°C, 70% r.h., 16 h:8 h light:dark) and offered to the insects at a pre-flowering stage, when about 5–6 weeks old. The insects were kept on their host plants in large mesh cages (60 × 60 × 60 cm) at room temperature and 16 h:8 h light:dark until the last larval instar, the eonymphs, crawled into the soil. Pots with soil and eonymphs were removed to containers with a gauze-lid. Once adults emerged, they were collected and kept in groups of up to 10 adults in Petri dishes (9 cm diameter) lined with filter paper and a source of honey-water mixture (1:50) in a refrigerator until use.

### Clerodanoid Exposure

Within 2 days after adult hatching, adult males and females were individually placed in small Petri dishes (5 cm diameter) lined with slightly moistened filter paper and placed at room temperature. Throughout the experiment, we did not further consider the sex of the animals but randomly distributed males and females into all treatments. They were provided with the honey-water mixture and half of the individuals were offered a small piece (1 × 1 cm) of a leaf of *Ajuga reptans* (Lamiaceae). Plants used for this experiment were kept outside in pots and were flowering. Adults usually start within less than 20 min to voraciously “nibble” on the leaves (Müller, personal observation) and take up clerodanoid compounds in that way (Nishida et al., 2004; Paul et al., 2021). We refer to this experimental manipulation as “exposure treatment.” After 48 h, the leaf was removed from the Petri dish and the bioassay was initiated (see below). Even though we do not have a direct quantification of the amount of compounds taken up after nibbling on the leaves, we know that nibbling on *A. reptans* leaves for a short time leads to qualitative changes in the chemical profiles of adults. After such exposure, adults become attractive to their conspecifics, which exhibit a nibbling behavior only on exposed but not on non-exposed conspecifics (Paul et al., 2021). We observed this behavior toward insects exposed to clerodanoids in our experiment, but not toward insects of the non-exposed treatment.

### Culture of *Beauveria bassiana* and Condidia Suspension

A *Beauveria bassiana* strain (KVL 03-122) was collected from an agroecosystem in Denmark (Meyling et al., 2009) isolated from *Leptoterna dolobrata* (Homoptera: Miridae). It was kept at −80°C in a culture collection at University of Copenhagen before cultivation. Isolates were cultivated on quarter-strength Sabouraud Dextrose Agar + Yeast 10% (SDAY) and incubated for 10 days at 23°C to allow for sporulation. Conidia were collected by scraping the surface of the culture with a sterile loop and transferred in sterile 1 ml phosphate buffered saline (PBS) with 0.05% of Triton-X. The resulting solution was centrifuged twice at 23°C, 4000 rpm for 3.5 min and the supernatant discarded in order to remove agar and hyphae. The pellet was resuspended in 1 ml PBS/Triton-X 0.05%, and the conidia concentration assessed with a hemocytometer (Neubauer Improved). The concentration of the inoculum was adjusted through serial dilutions before exposing the adults of *A. rosae*.

We chose to expose the sawflies to 2.10^7^, 2.10^8^, and 2.10^9^ conidia/ml. In a preliminary assay, 2.10^6^ conidia/ml did not cause any mortality significantly different from control insects (Supplementary Figure S1). We, therefore, chose to focus our sampling efforts on the three aforementioned inoculum concentrations.

After each infection bout (replicate), the germination rate of the inoculum was assessed by plating 100 μl of a 10^5^ conidia/ml solution on quarter strength SDAY 10% plates. After incubation for 24 h at 23°C, the germination of 3 × 100 conidia was counted to ensure that the germination rate or the inoculum was higher than 90%.

We chose the strain KVL 03-122 based on previous bioassays performed on *Tenebrio molitor* (Coleoptera: Tenebrionidae), which showed that KVL 03-122 was one of the most virulent strains we had access to, maybe because of its fast germination on the insect cuticle (unpublished data). Its host range is unknown but is likely to be broad, since it infects species of different orders, including beetles and mirids. Whether it has been in contact with populations of *A. rosae* in the field is unknown, but the ecology of *B. bassiana* makes the existence of an adaptation to the latter unlikely (Ortiz-Urquiza and Keyhani, 2013). Another strain, KVL 03-144, showing an intermediate virulence to *T. molitor*, did not cause a mortality significantly different from control animals in a preliminary experiment on *A. rosae* despite the relatively high concentration of the inoculum (2.10^8^ conidia/ml, Supplementary Figure S2).

### Bioassay

Adults with or without clerodanoid exposure were transferred individually from their Petri dishes to medicine cups (Carl Roth, Karlsruhe, Germany) containing 2 × 2 cm filter paper dipped in a filter-sterilized solution of honey in distilled water (1:50 v:v).

One microliter of conidia suspension of a given concentration was applied with a micropipette laterally where the legs are connected to the thorax, in order to facilitate the adsorption of the droplet during the movement of the adults. As a control, individuals received an application of PBS/Triton-X 0.05% only (referred to as “Triton” treatment). Care was taken that the whole droplet was adsorbed on the cuticle before including each individual in the experiment and closing the lid of the medicine cup. The medicine cups of a whole replicate were then kept in an incubator at 20°C and with a natural photoperiod. Every 2nd day, we transferred the individuals into a clean medicine cup with a fresh filter paper soaked in honey water. The experiments were carried out between April and May 2020. The day of inoculation was counted as day 0, and we refer to this experimental manipulation as “inoculation treatment.” Per treatment combination, between 17 and 34 individuals were set up (Table 1).

**Table 1:** Number of adult sawflies, which where either exposed or not to the plant *Ajuga reptans*, for uptake of clerodanoids, and then inoculated with different concentrations of *B. bassiana* conidia.

Inoculation treatment in number of conidia per ml	Exposure treatment	
	Non clerodanoids	Clerodanoids
Triton	32	34
2.10^7^	20	19
2.10^8^	17	22
2.10^9^	19	25

Individual sawflies were checked daily for movement, in which case, they were scored as alive. On the contrary, individuals lying at the bottom of the cup and showing no leg movement, or individuals showing a paralyzed and dehydrated look (characteristic of the death by *B. bassiana*) were scored as dead. Dead individuals were transferred into a new sterile medicine up and kept with a 3 × 3 cm piece of filter paper dipped in filter-sterilized distilled water, and kept in similar conditions. We checked daily for the re-emergence of the mycelium from the cadavers for 20 days.

Each fungal inoculation treatment concentration was performed twice in two separate inoculation sessions (replicates). Due to the number of insects we could obtain for each inoculation session, we could test only two concentrations simultaneously, alongside the corresponding Triton control. Therefore, the number of insects is higher in the Triton inoculation treatment.

### Statistics

All statistical analyses and graphical representations were performed with the R software (R Core Team, 2013).

The survival of *A. rosae* when inoculated with different concentrations of conidia showed non-proportional hazards. We analyzed the survival data with an accelerated failure time model fitted for a Weibull distribution with the “survreg” function of the “survival” package (Therneau and Grambsch, 2000; Therneau, 2015). Between-group comparisons were performed using the method of contrasts (Crawley, 2007).

Regarding the proportion of sawfly individuals from which we could see mycelium germinating on the external surface of the cadaver, we analyzed presence or absence of mycelium on the cadaver with a generalized linear model (GLM) fitted for a binomial distribution, with presence or absence of mycelium being the response variable, and the clerodanoid exposure (or no exposure) and concentration of the inoculum of conidia as explanatory variables. Since the adults inoculated with Triton never showed any mycelial growth on their surface after death, we excluded them from the analysis.

For both analyses, we started with the most complex models including two-way interactions between the inoculation and the exposure treatments as well as the inoculation session as a frailty term in the survival analysis, and as a random factor. We performed model selection by comparing Akaike’s information criterion (AIC) of the full models and all possible nested models, including the null one. We kept as optimal models the ones with the lowest AICs (Akaike, 1976). The figures were made with the “ggplot2” and “survminer” packages (Wickham, 2016; Kassambara and Kosinski, 2018).

## Results

### Survival of *Athalia rosae* Adults to *Beauveria bassiana* KVL 03-122 and in Depedence of Clerodanoid Exposure

**Figure 1:**
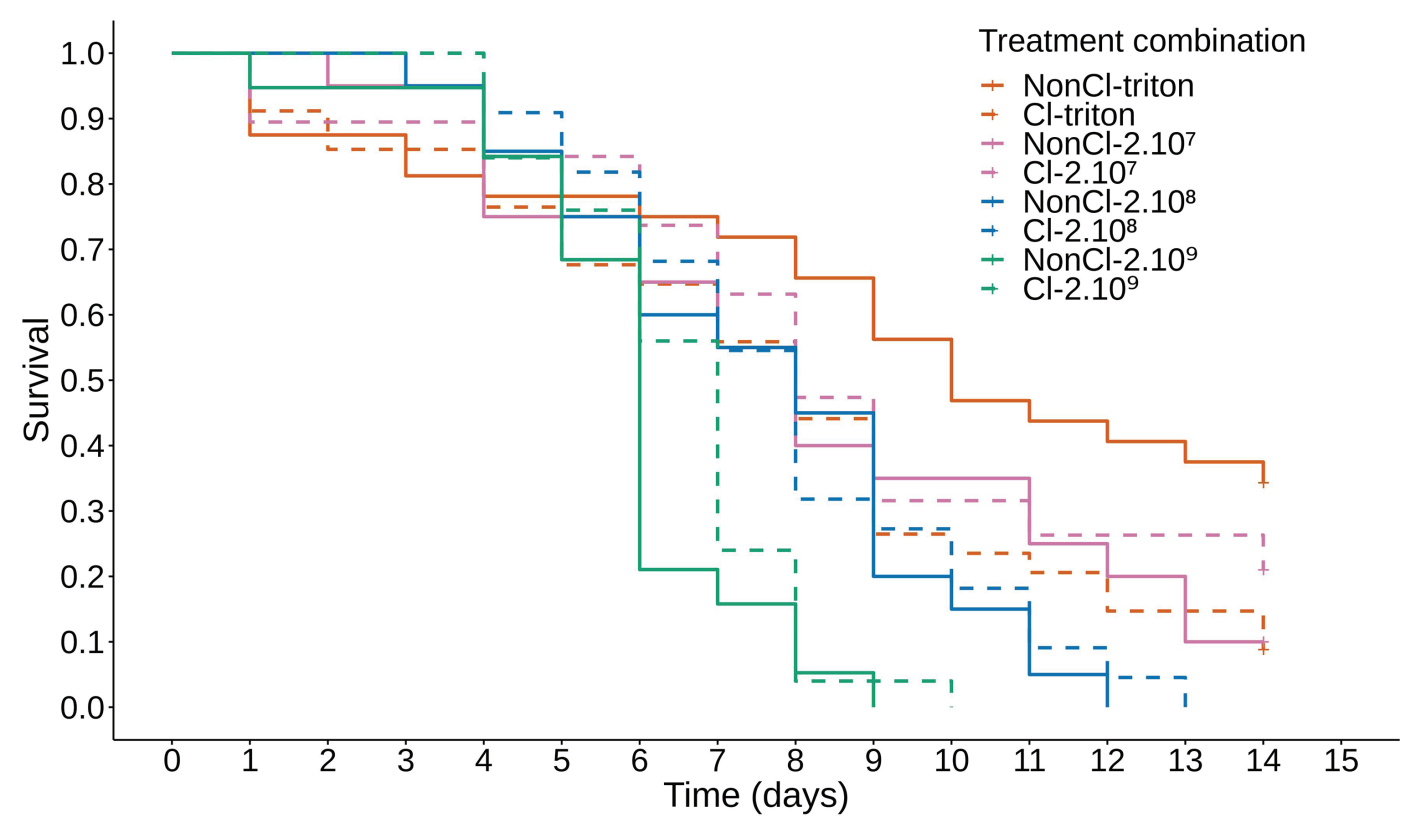
Survival of *Athalia rosae* adults inoculated with *Beauveria bassiana* and depending on clerodanoid exposure. Kaplan-Meier curve showing the proportion of live individuals over 14 days of *A. rosae* adults inoculated with several concentrations of conidia of *B. bassiana* strain KVL 03-122. Orange lines: 0.05% Triton X-PBS (triton); pink lines: 2.10^7^; blue lines: 2.10^8^; green lines: 2.19^9^ conidial/ml. Dashed lines represent adults allowed to take up clerodanoids (Cl) from a leaf of *Ajuga reptans* prior to inoculation (NonCl); survival of adults treated with Cl-triton was significantly different from those of the NonCl-triton treatment. There was no difference between survival of Cl and NonCl individuals in the other inoculation treatments with different concentrations of *B. bassiana*. Survival decreased with increasing concentrations of the inoculation treatment. See Supplementary Table S3 for *post hoc* comparisons.

The mortality of the experimental sawflies population showed a clear dose response to the concentration of conidia present in the inoculum, with the longevity of the sawflies decreasing with increasing concentrations of the inoculum between the treatment groups (Figure 1; Supplementary Table S3 for *post hoc* comparisons).

The effect of clerodanoid exposure on the survival of *A. rosae* adults over 14 days differed depending on the inoculation with *B. bassiana* conidia (inoculation treatment*exposure treatment: deviance = 10.13; *p* = 0.018; df = 9,182). Individuals that were exposed to clerodanoids prior to inoculation with the non-infectious control (0.05% Triton X-PBS) died after a shorter time compared to those not exposed to clerodanoids. In contrast, when the individuals were inoculated with conidia of *B. bassiana*, there was no difference between individuals exposed to clerodanoids or not (Figure 1; Supplementary Table S3 for *post hoc* comparisons).

### Sporulation of *Beauveria bassiana* From Adult Sawfly Cadavers

**Figure 2:**
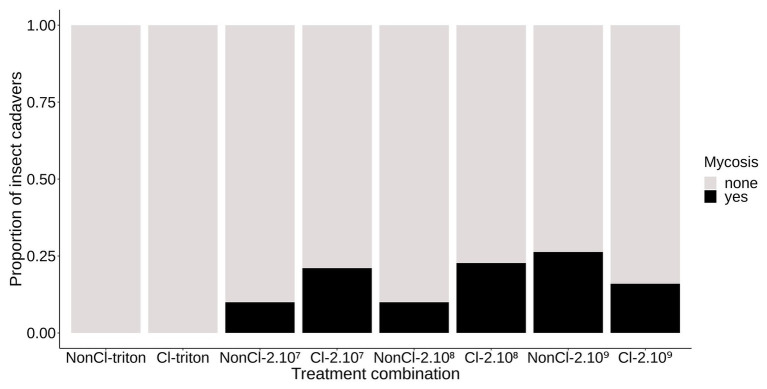
Mycelial colonization of the cadavers of *A. rosae*. Stacked bar graph representing the proportion of *A. rosae* cadavers showing mycelial colonization, i.e., mycosis (yes) or not (none) by *B. bassiana* KVL 03-122 depending on the inoculation and exposure treatments: Non-Cl, no clerodanoids exposure, Cl, clerodanoids exposure; triton, 2.10^7^, 2.10^8^, and 2.10^9^ represent the inoculation control and the inoculum concentration in conidial/ml. Neither the inoculation nor the clerodanoids exposure treatments significantly affected the proportion of cadavers showing mycelial colonization.

We found a very low incidence of mycelial growth and sporulation from cadavers of *A. rosae* that had been inoculated with different concentrations of conidia. The proportion of cadavers from which fungal sporulation occurred was between 10 and 26% in the different experimental groups (Figure 2). Among the adult sawflies inoculated with conidia, neither the clerodanoid exposure nor the concentration of conidia of the inoculum explained the incidence of mycelial growth on the cadavers (inoculation treatment∗exposure treatment: deviance = 2.51; *p* = 0.28; df = 5,119; inoculation treatment: deviance = 0.4; *p* = 0.82; df = 2,122; exposure treatment: deviance = 0.43; *p* = 0.51; df = 1,123). As expected, no mycelium germinated on the external surface of adults not exposed to conidia.

## Discussion

This study aimed at assessing the virulence of a *B. bassiana* strain against adult sawflies of *A. rosae*, which are known to expose themselves to clerodanoids in their natural environment. Manipulating both the amount of conidia the sawfly individuals received and whether or not they were allowed to take up clerodanoids enabled us to gather data on both the sawfly-fungus interaction and the potential benefit of clerodanoid uptake.

The inoculum concentrations of *B. bassiana* we had to use to induce mortality significantly higher than in control individuals of *A. rosae* were relatively high compared to lethal concentrations used in other insect-*B. bassiana* interactions (Maistrou et al., 2018, 2020). This indicates that adults of *A. rosae* have a rather high baseline resistance against this fungus. This species is indeed often confronted with entomopathogenic fungi, as the larvae bury themselves in the soil, where they pupate. Thus, also the emergence of the young adults happens in the soil, where entomopathogenic fungi are omnipresent (Clifton et al., 2015). This constant pressure could explain the evolution of a high level of constitutive resistance against such pathogens in *A. rosae*.

Another surprising result is that very few cadavers supported the re-emergence of our strain of *B. bassiana*. The mycosis developing at the surface of the insect cadaver is often used by invertebrate pathologists as a means of confirming that the insect died of the inoculated fungus instead of dying of a co-occuring opportunistic infection (Goettel and Inglis, 1997). In this context, it may seem that very few sawflies directly died of our strain of *B. bassiana*. However, this is unlikely, because their survival showed a clear dose-response to the concentration of conidia in the inoculum. Combined with the fact that we needed to inoculate the sawflies with a high dose of conidia, this confirms, as proposed by Maistrou et al. (2020), that the ability of one strain of *B. bassiana* to develop mycosis on the cadaver of the host is a fitness trait, which is correlated with other virulence traits, such as its ability to kill the host. It has been proposed that the ability to cause mycosis on the insect cadaver relies strongly on the ability to outcompete bacterial growth after host death (Fan et al., 2017). More experiments are required to explore this hypothesis in *A. rosae* adults in order to know whether this can explain a significant reduction in lifespan of the individuals compared to control with a low occurrence of mycosis on cadavers.

Interestingly, we observed no protective effect of the prior exposure of individual sawflies to clerodanoids, neither in the presence, nor absence of the fungus. This is an unexpected result considering that some clerodanoids show antifungal activity (reviewed by Li et al., 2016; Qing et al., 2017). The lack of a protective effect in this interaction suggests that the *in vitro* antimicrobial activity reported for some clerodanoids does not necessarily translate into a better protection against a potential pathogen *in vivo*. The activity may thus depend on the specific structure of the clerodanoids or other factors that modulate the insect’s physiology when incorporating compounds from *A. reptans*. There was moreover a slightly negative effect of clerodanoid uptake on survival of sawflies in the absence of fungal inoculation, as these insects showed a lower survival than the control. This corroborates results found by Kubo et al. (1982) who found insecticidal activities of two ajugarins, which are clerodanoids from *Ajuga* plants. Clerodanoids have been shown to affect insect physiology both by cuticular contact and feeding (Abbaszadeh et al., 2012). Thus, clerodanoid uptake may also come with some costs.

In contrast to the slightly negative effect of clerodanoids on adult survival, clerodanoid uptake has been shown to increase the mating success of female *A. rosae* (Amano et al., 1999), indicating a fitness benefit. A trade-off between reproduction and longevity has been observed in several insect species (Ellers, 1995; Hunt et al., 2006), and pharmacophagous uptake of plant compounds may have both costs and benefits (Erb and Robert, 2016). Moreover, in our study, the sawflies were not allowed to mate and had no opportunity to lay any eggs, as no host plant was provided. This may have affected their longevity as well. The longevity of the control animals treated with 0.05% Triton-X only was highly similar to the life-span observed for *A. rosae* in other studies, in which adult longevity had been investigated in dependence of the larval host plant quality (Müller and Sieling, 2006: on average 14 days; Bandeili and Müller, 2010: ranging from 9 to 17 days). Considering this relatively short adult lifespan of this species, the ability to reproduce during this time window is crucial for the individuals. Further studies should assess the effects of clerodanoid uptake on other life history traits such as fecundity and its relationship to longevity.

A negative effect of clerodanoids on the survival was not visible when the insects were inoculated with conidia, indicating that the deleterious effect of *B. bassiana* overwrites a potential negative effect of clerodanoids on *A. rosae*. In our system, we could not control for the amount of clerodanoids taken up by the adult sawflies during exposure. However, as *A. rosae* start within a very short time to nibble on leaves and as they had access to a leaf for 48 h, we can ensure that all insects had sufficient chance to take up clerodanoids. This was confirmed by the characteristic nibbling behavior conspecifics exhibited toward insects of the clerodanoids-exposed treatment. Adult sawflies could have either incorporated plant compounds and/or spread some over the surface of their cuticle when contacting the leaves or while cleaning themselves (Paul et al., 2021). Further experiments are needed to determine the exact location of clerodanoids in and/or on the individual sawflies.

Overall, our data suggest that the behavior of clerodanoid uptake did not evolve as a means to increase survival to an exposure to entomopathogenic fungi, at least not toward this *B. bassiana* strain. Since its deleterious effect on control individuals represents a cost which might be non-negligible in natural populations, we can assume that other benefits selected for the evolution and persistence of this trait in *A. rosae*. The above-mentioned benefit in mating and a reduction in the predation by birds and reptiles in insects which had taken up clerodanoids are some of these benefits (Nishida and Fukami, 1990).

To conclude, our study did not reveal our strain of *B. bassiana* to be a good candidate as a biocontrol agent of *A. rosae*, since it was only able to cause significant mortality at high doses. More importantly, our strain was not able to cause mycosis on the cadavers. This means that in the eventuality of a massive release of conidia of this strain on *A. rosae* populations, the fitness of the fungus would be almost zero, since the persistence of *B. bassiana* in the environment has been shown to rely mainly on conidia production (Vänninen, 1995). In case of a massive release of conidia for biocontrol, the lack of their persistence in the environment would prevent the infection of non-target species, as well as prevent the disruption of the microbial community of the soil, or select for resistance in pest populations. A suitable strategy may be to test other strains of *B. bassiana* in order to identify more virulent ones or some in which virulence may be decoupled from the ability to cause mycosis. Another lead would be to combine *B. bassiana* with cuticle-disrupting agents, like entomopathogenic nematodes or diatomaceous earth, which are known to have a synergistic effect with *B. bassiana* on host mortality (Barberchek and Kaya, 1990; Riasat and Wakil, 2011). These agents might increase host death without enabling the fungus to compete against bacteria after host death. Finally, the pharmacophagous uptake of clerodanoids did not reveal to improve resistance of *A. rosae* individuals against this fungus but may be an effective self-medication against other microorganisms and pathogens.

## Data Availability Statement

The raw data supporting the conclusions of this article is provided in the Supplementary Table S4.

## Author Contributions

CZ, JK, and CM conceived the experiments. CZ, LL, RR, and IM performed the experiments. CZ analyzed the data. CZ and CM wrote the manuscript, with inputs from LL, RR, and JK. CM and JK acquired funding. All authors contributed to the article and approved the submitted version.

### Conflict of Interest

The authors declare that the research was conducted in the absence of any commercial or financial relationships that could be construed as a potential conflict of interest.

## References

[ref1] Abbaszadeh G. Srivastava C. Walia S. (2012). Insect growth inhibitory activity of clerodane diterpenoids isolated from *Clerodendron infortunatum* L. on the cotton bollworm, *Helicoverpa armigera* (Hubner). Natl. Acad. Sci. Lett. 35, 457–464. 10.1007/s40009-012-0077-z PMC4206234 25373176

[ref2] Abbott J. (2014). Self-medication in insects: current evidence and future perspectives. Ecol. Entomol. 39, 273–280. 10.1111/een.12110

[ref3] Akaike H. (1976). “Canonical correlation analysis of time series and the use of an information criterion” in System identification advances and case studies. eds. Mehra R. K. Lainiotis D. G. (New York, NY, USA: Academic Press Inc.).

[ref4] Amano T. Nishida R. Kuwahara Y. Fukami H. (1999). Pharmacophagous acquisition of clerodendrins by the turnip sawfly (*Athalia rosae ruficornis*) and their role in the mating behaviour. Chemoecology 9, 145–150. 10.1007/s000490050046

[ref5] Bandeili B. Müller C. (2010). Folivory versus florivory—adaptiveness of flower-feeding. Naturwissenschaften 97, 79–88. 10.1007/s00114-009-0615-9, PMID: 19826770

[ref6] Barberchek M. E. Kaya H. K. (1990). Interactions between Beauveria bassiana and the entomogenous nematodes, Steinernema feltiae and Heterorhabditis heliothidis. J. Invertebr. Pathol. 55, 225–234. 10.1016/0022-2011(90)90058-E

[ref7] Boivin G. Hance T. Brodeur J. (2012). Aphid parasitoids in biological control. Can. J. Plant Sci. 92, 1–12. 10.4141/cjps2011-045

[ref8] Bonadies E. Wcislo W. T. Gálvez D. Hughes W. Fernández-Marín H. (2019). Hygiene defense behaviors used by a fungus-growing ant depend on the fungal pathogen stages. Insects 10:130. 10.3390/insects10050130, PMID: 31060310 PMC6572560

[ref9] Bozov P. Girova T. Prisadova N. Hristova Y. Gochev V. (2015). Antimicrobial activity of neo-clerodane diterpenoids isolated from Lamiaceae species against pathogenic and food spoilage microorganisms. Nat. Prod. Commun. 10, 1797–1800. 10.1177/1934578X1501001101, PMID: 26749799

[ref10] Clifton E. H. Jaronski S. T. Hodgson E. W. Gassmann A. J. (2015). Abundance of soil-borne entomopathogenic fungi in organic and conventional fields in the midwestern USA with an emphasis on the effect of herbicides and fungicides on fungal persistence. PLoS One 10:e0133613. 10.1371/journal.pone.0133613, PMID: 26191815 PMC4507996

[ref11] Cotes B. Rämert B. Nilsson U. (2018). A first approach to pest management strategies using trap crops in organic carrot fields. J. Crop Prot. 112, 141–148. 10.1016/j.cropro.2018.05.025

[ref12] Crawley M. J. (2007). The R book. Chichester, England: John Wiley & Sons.

[ref13] Daniel R. A. Ion M. (2019). The protection of the rapeseed crop against the attack of *Athalia rosae* in the S-E of Boianului plain. Annals of the University of Craiova, Agriculture, Montanology, Cadastre Series Vol. XLIX.

[ref14] Ellers J. (1995). Fat and eggs: an alternative method to measure the trade-off between survival and reproduction in insect parasitoids. Neth. J. Zool. 46, 227–235. 10.1163/156854295X00186

[ref15] Erb M. Robert C. A. M. (2016). Sequestration of plant secondary metabolites by insect herbivores: molecular mechanisms and ecological consequences. Curr. Opin. Plant. Sci. 14, 8–11. 10.1016/j.cois.2015.11.005, PMID: 27436640

[ref16] Fan Y. Liu X. Keyhani N. O. Tang G. Pei Y. Zhang W.. (2017). Regulatory cascade and biological activity of *Beauveria bassiana* oosporein that limits bacterial growth after host death. Proc. Natl. Acad. Sci. U. S. A. 114, E1578–E1586. 10.1073/pnas.1616543114, PMID: 28193896 PMC5338512

[ref17] Ferro D. N. Morzuch B. J. Margolies D. (1983). Crop loss assessment of the Colorado potato beetle (Coleoptera: Chrysomelidae) on potatoes in Western Massachusetts. J. Econ. Entomol. 76, 349–356. 10.1093/jee/76.2.349

[ref18] Glare T. R. O’Callaghan M. (2000). *Bacillus thuringiensis*: Biology, ecology and safety. 1st Edn. New York, NY, USA: Wiley.

[ref19] Goettel M. S. Inglis G. D. (1997). “Chapter V-3—Fungi: hyphomycetes” in Manual of techniques in insect pathology. ed. Lacey L. A. (San Diego, CA: Academic Press), 213–249.

[ref20] Hunt J. Jennions M. D. Spyrou N. Brooks R. (2006). Artificial selection on male longevity influences age dependent reproductive effort in the black field cricket *Teleogryllus commodus*. Am. Nat. 168, E72–E86. 10.1086/506918, PMID: 16947102

[ref21] Hunt D. W. A. Vernon R. S. (2001). Portable trench barrier for protecting edges of tomato fields from Colorado potato beetle (Coleoptera: Chrysomelidae). J. Econ. Entomol. 94, 204–207. 10.1603/0022-0493-94.1.204, PMID: 11233114

[ref22] Inceoglu A. B. Kamita S. G. Hinton A. C. Huang Q. Severson T. F. Kang K. D.. (2001). Recombinant baculoviruses for insect control. Pest Manag. Sci. 57, 981–987. 10.1002/ps.393, PMID: 11695193

[ref23] Inglis G. D. Goettel M. S. Butt T. M. Strasser H. (2001). “Use of hyphomycete fungi for managing insect pests” in Fungi as biological agents: Progress, problems and potential. eds. Butt T. M. Jackson C. W. Magan N. (Wallingford, UK: CABI Publishing), 23–69.

[ref24] Kamangar S. Gharali B. Kaihanian A. A. Ebrahimi E. (2010). Identification of natural enemies of *Athalia rosae* and investigation on effects of these agents on the control of the pest in Kurdistan province.

[ref25] Kassambara A. Kosinski M. (2018). Survminer: Drawing Survival Curves using “ggplot2.” R Package Version 0.4.2. 2018. Available at: https://CRAN.R-project.org/package=survminer (Accessed July 20, 2020).

[ref26] Kubo I. Klocke J. A. Miura l. Fukuyama Y. (1982). Structure of Ajugarin IV. J. Chem. Soc. Chem. Commun. 11, 618–619.

[ref27] Kuhar T. P. Youngman R. R. Laub C. A. (2000). Alfalfa weevil (Coleoptera: Curculionidae) population dynamics and mortality factors in Virginia. Environ. Entomol. 29, 1295–1304. 10.1603/0046-225X-29.6.1295

[ref28] Lacey L. A. Grzywacz D. Sapiro-Ilan D. I. Frutos R. Brownbridge M. Goettel M. S. (2015). Insect pathogens as biological control agents: back to the future. J. Invertebr. Pathol. 132, 1–41. 10.1016/j.jip.2015.07.009, PMID: 26225455

[ref29] Laznik Ž. Tóth T. Lakatos T. Vidrih M. Trdan S. (2010). Control of the Colorado potato beetle (*Leptinotarsa decemlineata* [Say]) on potato under field conditions: a comparison of the efficacy of foliar application of two strains of *Steinernema feltiae* (Filipjev) and spraying with thiametoxam. J. Plant. Dis. Prot. 117, 129–135. 10.1007/BF03356348

[ref30] Li R. Morris-Natschke S. L. Lee K. H. (2016). Clerodane diterpenes: sources, structures, and biological activities. Nat. Prod. Rep. 33, 1166–1226. 10.1039/c5np00137d, PMID: 27433555 PMC5154363

[ref31] Lombaert E. Boll R. Lapchin L. (2006). Dispersal strategies of phytophagous insects at a local scale: adaptive potential of aphids in an agricultural environment. BMC Evol. Biol. 6:75. 10.1186/1471-2148-6-75, PMID: 17014710 PMC1622755

[ref32] Maistrou S. Natsopoulou M. E. Jensen A. B. Meyling N. V. (2020). Virulence traits within a community of the fungal entomopathogen Beauveria: associations with abundance and distribution. Fungal Ecol. 48:100992. 10.1016/j.funeco.2020.100992

[ref33] Maistrou S. Paris V. Jensen A. B. Rolff J. Meyling N. V. Zanchi C. (2018). A constitutively expressed antifungal peptide protects *Tenebrio molitor* during a natural infection by the entomopathogenic fungus *Beauveria bassiana*. Dev. Comp. Immunol. 86, 26–33. 10.1016/j.dci.2018.04.015, PMID: 29698631

[ref34] Meyling N. V. Lübeck M. Buckley E. P. Eilenberg J. Rehner S. A. (2009). Community composition, host-range and genetic structure of the fungal entomopathogen *Beauveria* in adjoining agricultural and semi-natural habitats. Mol. Ecol. 18, 1282–1293. 10.1111/j.1365-294X.2009.04095.x, PMID: 19226319

[ref35] Meyling N. V. Pell J. K. (2006). Detection and avoidance of an entomopathogenic fungus by a generalist insect predator. Ecol. Entomol. 31, 162–171. 10.1111/j.0307-6946.2006.00781.x

[ref36] Müller C. Sieling N. (2006). Effects of glucosinolate and myrosinase levels in *Brassica juncea* on a glucosinolate-sequestering herbivore – and vice versa. Chemoecology 16, 191–201. 10.1007/s00049-006-0347-7

[ref37] Nishida R. Fukami H. (1990). Sequestration of distasteful compounds by some pharmacophagous insects. J. Chem. Ecol. 16, 151–164. 10.1007/BF01021276, PMID: 24264904

[ref38] Nishida R. Kawai K. Amano T. Kuwahara Y. (2004). Pharmacophagous feeding stimulant activity of neo-clerodane diterpenoids for the turnip sawfly, *Athalia rosae ruficornis*. Biochem. Syst. Ecol. 32, 15–25. 10.1016/S0305-1978(03)00160-1

[ref39] Ohnesorge B. (1979). Beobachtungen zur Biologie der Rubsenblattwespe *Athalia rosae* L. (Hym., Tenthredinidae). Anzeiger fur Schadlingskunde Pflanzenschutz Umweltschutz 52, 70–73. 10.1007/BF01903535

[ref40] Opitz S. E. W. Boevé J. -L. Nagy Z. T. Sonet G. Koch F. Müller C. (2012). Host shifts from Lamiales to Brassicaceae in the sawfly genus *Athalia*. PLoS One 7:e33649. 10.1371/journal.pone.0033649, PMID: 22485146 PMC3317781

[ref41] Ortiz-Urquiza A. Keyhani N. O. (2013). Action on the surface: entomopathogenic fungi versus the insect cuticle. Insects 4, 357–374. 10.3390/insects4030357, PMID: 26462424 PMC4553469

[ref42] Paul S. C. Dennis A. B. Tewes L. -J. Freidrichs J. Müller C. (2021). Consequences of pharmacophagous uptake from plants and conspecifics in a sawfly elucidated using chemical and molecular techniques. bioRxiv [preprint] 10.1101/2021.02.09.430406

[ref43] Pedrini N. Ortiz-Urquiza A. Huarte-Bonnet C. Zhang S. Keyhani N. O. (2013). Targeting of insect epicuticular lipids by the entomopathogenic fungus *Beauveria bassiana*: hydrocarbon oxidation within the context of a host-pathogen interaction. Front. Microbiol. 15, 4–24. 10.3389/fmicb.2013.00024, PMID: 23422735 PMC3573267

[ref44] Qing X. Yan H. Ni Z. Y. Vavricka C. Zhang M. Shi Q.. (2017). Chemical and pharmacological research on the plants from genus *Ajuga*. Heterocycl. Commun. 23, 245–268. 10.1515/hc-2017-0064

[ref46] Rangel D. E. Anderson A. J. Roberts D. W. (2008). Evaluating physical and nutritional stress during mycelial growth as inducers of tolerance to heat and UV-B radiation in *Metarhizium anisopliae* conidia. Mycol. Res. 112, 1362–1372. 10.1016/j.mycres.2008.04.013, PMID: 18938068

[ref45] R Core Team (2013). R: A language and environment for statistical computing. R Foundation for statistical Computing, Vienna, Austria. Available at: http://www.R-project.org/ (Accessed March 15, 2018).

[ref47] Riasat T. Wakil W. (2011). Effect of Beauveria bassiana mixed with diatomaceous earth on mortality, mycosis and sporulation of *Rhyzopertha dominica* on stored wheat. Phytoparasitica 39, 225–231. 10.1007/s12600-011-0164-6

[ref48] Riggert E. (1939). Untersuchungen über die Rübenblattwespe *Athalia colobri* Christ (*A. spinarum* F.). Z. Angew. Entomol. 26, 462–516. 10.1111/j.1439-0418.1939.tb01575.x

[ref49] Santa H. S. Santa O. R. Brand D. Vandenberghe L. P. Soccol C. R. (2005). Spore production of Beauveria bassiana from agro-industrial residues. Braz. Arch. Biol. Technol. 48, 51–60. 10.1590/S1516-89132005000400007

[ref50] Sáringer G. Fodor A. Nadasy M. Lucskai A. Georgis R. (1996). Possibilities of biological control using entomopathogenic nematodes against *Leptinotarsa decemlineata* L. and *Athalia rosae* L. larvae. Mededelingen-Faculteit Landbouwkundige en Toegepaste Biologische Wetenschappen Universiteit Gent (Belgium).

[ref51] Sawa M. Fukunaga A. Naito T. Oishi K. (1989). Studies on the sawfly, *Athalia rosae* (Insecta, Hymenoptera, Tenthredinidae) I. general biology. Zool. Sci. 6, 541–547.

[ref52] Sharma S. Kooner R. Arora R. (2017). “Insect pests and crop losses” in Breeding insect resistant crops for sustainable agriculture. eds. Arora R. Sandhu S. (Singapore: Springer).

[ref480] Therneau T. M. A. (2015). Package for Survival Analysis in S. Package Version 2.38. Available at: https://CRAN.R-project.org/package=survival (Accessed April 10, 2020).

[ref481] Therneau T. M. Grambsch P. M. (2000). Modeling Survival Data: Extending the Cox Model. New York, NY: Springer.

[ref53] Vallat A. Dorn S. (2005). Changes in volatile emissions from apple trees and associated response of adult female codling moths over the fruit-growing season. J. Agric. Food Chem. 53, 4083–4090. 10.1021/jf048499u, PMID: 15884843

[ref54] Vänninen I. (1995). Distribution and occurrence of four entomopathogenic fungi in Finland: effect of geographical location, habitat type and soil type. Mycol. Res. 100, 93–101. 10.1016/S0953-7562(96)80106-7

[ref55] Wickham H. (2016). ggplot2: Elegant graphics for data analysis. New York, NY, USA: Springer.

[ref56] Zalucki M. P. Shabbir A. Silva R. Adamson D. Shu-Sheng L. Furlong M. J. (2012). Estimating the economic cost of one of the world’s major insect pests, *Plutella xylostella* (Lepidoptera: Plutellidae): just how long is a piece of string? J. Econ. Entomol. 105, 1115–1129. 10.1603/EC12107, PMID: 22928287

