# Supplementary material for: Survival of the Sawfly Athalia rosae Upon Infection by an Entomopathogenic Fungus and in Relation to Clerodanoid Uptake
Source: Front Physiol. 2021 Mar 24;12:637617. doi: 10.3389/fphys.2021.637617 (PMC8024555; doi:10.3389/fphys.2021.637617)
Supplement: Supplementary file 1 [file Data_Sheet_1.pdf]

## Supplementary Material

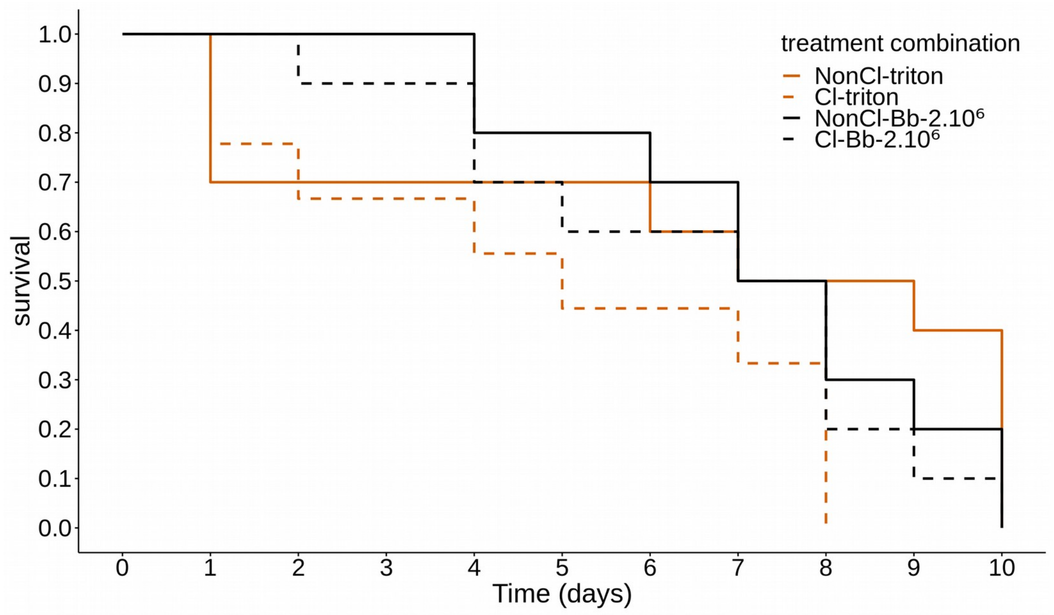


**Figure S1: Survival of *A. rosae* wasps inoculated with *B. bassiana* strain KVL-03-122 and in dependence of clerodanoid exposure.** Kaplan-Meier curve showing the survival of *A. rosae* wasps inoculated or not with *B. bassiana* (Bb) strain KVL-122. Orange lines: 0.05 % Triton X-PBS (Cl-triton); black lines: $2.10^6$ conidial/mL of 0.05 % Triton X-PBS. Dashed lines represent wasps allowed to take up clerodanoids from a leaf of *Ajuga reptans* prior to inoculation, whereas plain lines represent the wasps that were not offered a leaf prior to inoculation. N = 10 per treatment combination over 1 replicate. The inoculation of $2.10^6$ conidia/mL did not affect the survival of the wasps compared to the Triton-inoculated control, since the null model was better supported than the full model with the inoculation*exposure treatment interaction as well as all derived models. We decided not to perform further bioassays with this inoculum concentration.

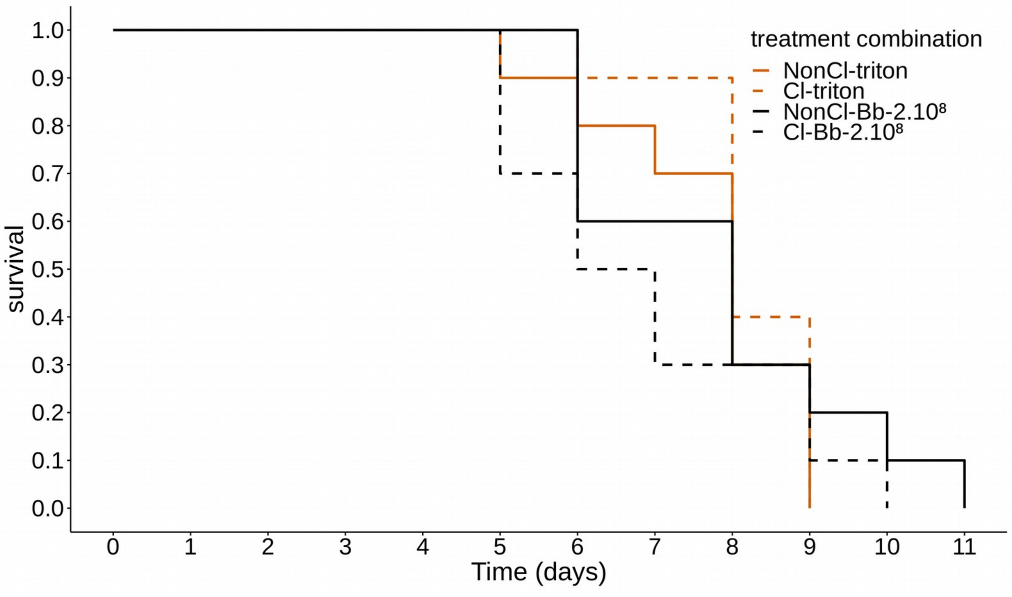


**Figure S2: Survival of *A. rosae* wasps inoculated with *B. bassiana* KVL-03-144 and in dependence of clerodanoid exposure.** Kaplan-Meier curve showing the survival of *A. rosae*

wasps inoculated or not with *B. bassiana* (Bb) strain KVL 03-144. Orange lines: 0.05 % Triton X-PBS (Cl-triton); black lines: $2.10^{8}$ conidial/mL of 0.05 % Triton X-PBS. Dashed lines represent wasps allowed to take up clerodanoids from a leaf of *Ajuga reptans* prior to inoculation, whereas plain lines represent the wasps that were not offered a leaf prior to inoculation. N = 10 per treatment combination over 1 replicate. The inoculation of $2.10^{8}$ conidia/mL did not affect the survival of the wasps compared to the Triton-inoculated control, since the null model was better supported than the full model with the inoculation*exposure treatment interaction as well as all derived models. Considering $2.10^{8}$ conidia/mL is a high inoculum concentration, we decided not to perform further bioassays with this strain.

| **Exposure Treatment** | | Triton | | $2.10^{7}$ condia/mL | | $2.10^{8}$ conidia/mL | | $2.10^{9}$ conidia/mL | |
|---|---|---|---|---|---|---|---|---|---|
| | **Inoculation Treatment** | control | clerodanoid | control | clerodanoid | control | clerodanoid | control | clerodanoid |
| Triton | control | | | | | | | | |
| | clerodanoid | **Z = -2.74<br>P = 0.006** | | | | | | | |
| $2.10^{7}$ Conidia/mL | control | **Z = -2.00<br>P = 0.045** | Z = 0.20<br>P = 0.85 | | | | | | |
| | clerodanoid | Z = -1.18<br>P = 0.24 | Z = 0.96<br>P = 0.34 | Z = 0.75<br>P = 0.45 | | | | | |
| $2.10^{8}$ Conidia/mL | control | **Z = -4.23<br>P = 2.4 e-05** | **Z = -2.53<br>P = 0.011** | **Z = -2.57<br>P = 0.012** | **Z = -3<br>P = 0.003** | | | | |
| | clerodanoid | **Z = -4.20<br>P = 2.7e-05** | **Z = -2.51<br>P = 0.012** | **Z = -2.64<br>P = 0.008** | **Z = -3.09<br>P = 0.002** | Z = -0.02<br>P = 0.991 | | | |
| $2.10^{9}$ Conidia/mL | control | **Z = -6.22<br>P = 4.9e-10** | **Z = -4.52<br>P = 6.3e-06** | **Z = -3.49<br>P = 4.9e-03** | **Z = -3.89<br>P = 9.9e-05** | **Z = -2.29<br>P = 0.022** | **Z = -2.62<br>P = 0.001** | | |
| | clerodanoid | **Z = -5.08<br>P = 3.9e-07** | **Z = -3.40<br>P = 6.8e-03** | **Z = -2.88<br>P = 0.004** | **Z = -3.34<br>P = 8.5e-03** | **Z = -2.5<br>P = 0.011** | **Z = -2.75<br>P = 0.005** | Z = 1.64<br>P = 0.101 | |

**Table S3 for post-hoc comparisons of the survival of *A. rosae* exposed to *B. bassiana* KVL 03-122.** Associated p values and estimates of the full model testing the effect of the clerodanoid exposure treatment and the conidia concentration in the inoculation treatment on the survival of *A. rosae* wasps over 14 days. Significant p values are presented in bold. The interaction between the exposure and inoculation treatments is significant. See also Figure 1.
